# Editorial: Pandemic, war and climate changes: the effect of these crises on individual and social well-being

**DOI:** 10.3389/fpsyg.2024.1473156

**Published:** 2024-08-26

**Authors:** Ciro Esposito, Immacolata Di Napoli, Bernd Roehrle, Caterina Arcidiacono

**Affiliations:** ^1^Department of Humanities, University of Foggia, Foggia, Italy; ^2^Department of Humanities, University of Naples Federico II, Naples, Campania, Italy; ^3^Department of Clinical Psychology and Psychotherapy, University of Marburg, Marburg, Hesse, Germany

**Keywords:** individual wellbeing, social wellbeing, COVID-19, uncertainty, flexibility and hope

From 2020 to the present, a series of events and crises have impacted people around the world, putting their physical and psychological health at risk. Indeed, individuals have faced and are encountering considerable stressors, starting with the COVID-19 pandemic, which began in March 2020, continuing with the invasion of Ukraine by Russia, which took place in February 2022 (the most immediate war in Europe, like many warlike conflicts existing simultaneously worldwide), and including the climate change, which is becoming increasingly evident, problematic, globally worrying, and posing an existential threat (Francescato, 2020; Arcidiacono, 2024; EFPA, 2024).

These crises, acting either singly or in an interrelated form, had strong effects on different kinds of wellbeing (e.g., life satisfaction) of people and their interpersonal relationships, negatively affecting their health (Röhrle, 2024). All of these stressors have been well analyzed in terms of their effects on wellbeing. Recently, questions have arisen about how these multiple stressors interact, for example, how COVID-19, the climate crisis, and economic and social conflicts interact with each other. They have had both direct and indirect impacts on relevant behaviors and health via physical, biological, and economic characteristics, social identification processes, action readiness, and self-efficacy.

Therefore, this Research Topic aimed to address the interaction among different global threats (COVID-19, the climate crisis, war, or other comprehensive stressors), life events, and hassles. Moreover, it was oriented toward multiple levels of influence, direct and indirect interactions of predictors, mediators, and moderators and to detect their overall influence on people's wellbeing (in a broader sense) and their interpersonal relationships.

The final objective was to detect and improve the best life strategies for organizing our lives and environments under these circumstances. Therefore, studies or projects focusing on possible intervention approaches that have assisted or could assist people in better facing the current emergencies, while promoting their individual and social wellbeing, were encouraged.

The contributions evaluated differences in the consequences of these events on the wellbeing of people, comparing different territorial areas or countries and evaluating the progress of people's psychological wellbeing over time.

Significant results, however, need a longer time perspective. Despite the urgency of these questions, the call for articles could only partially reach researchers who have focused strongly on COVID-19 and wellbeing. Thus, this Research Topic only partially provides an insight into the question of how COVID-19 affects wellbeing and which subjective processes are also involved as mediating factors.

Regardless of the issue of multiple crises, the thematic focus on wellbeing is also a key aspect of COVID-19 research. This focus is peculiar because COVID-19 has not only been predominantly investigated for its significance as a pathogenic phenomenon, but also for its psychological repercussions. Reviews and meta-analyses on the significance of wellbeing in the context of COVID-19 are rare (e.g., Esposito et al., 2021; Blasco-Belled et al., 2024). Although the compilation of articles in Research Topic focuses on COVID-19, it also contributes something enlightening to the question of wellbeing when people are confronted with stressors.

To achieve the objectives of this Research Topic, six articles have been included. As mentioned, despite the vast scope of the call regarding the effects of different types of contemporary crises on wellbeing, these articles are all focused on what is perhaps to be considered the turning point par excellence of our era, that is, the COVID-19 pandemic.

The topics of primary interest for Research Topic on “*Pandemic, war and climate changes: the effect of these crises on individual and social well-being*” include the effects of lockdown periods on people's psychological wellbeing, both in the immediate and long term, especially among students. University students, adolescents and young adults in general, appear to be one of the populations most adversely affected by the psycho-social effects of the COVID-19 pandemic and the resulting lockdown periods (Di Napoli et al., 2021; Marzana et al., 2021; Migliorini et al., 2021; Procentese et al., 2021; Novara et al., 2022).

The article “*Risk perception of coronavirus disease 2019 and career adaptability among college students: the mediating effect of hope and sense of mastery*” (Ding and Li) describes whether the risk perception of COVID-19 is associated with career adaptability directly and indirectly through hope and a sense of mastery in Chinese college students. Hope is seen as a close correlate of wellbeing. A questionnaire survey conducted among 594 Chinese college students, aged 16–25 years, assessed the risk perception of COVID-19, career adaptability, hope, and a sense of mastery. The research described the effects of three types of risk perception (susceptibility, severity, and uncontrollability), showing that susceptibility was negatively associated with career confidence, control, and curiosity; moreover, susceptibility indirectly affected career adaptability (including concern, confidence, control, and curiosity) through the sense of mastery; uncontrollability indirectly affected career concern through hope and it also indirectly affected career adaptability (including concern, confidence, control, and curiosity) through hope and the sense of mastery. In the meanwhile, the findings emphasize the role of hope and a sense of mastery in career adaptability, focusing on improving hope and a sense of mastery to promote college students' career development.

Moreover, Lin, in the article “*The influence mechanism underlying meaning in life on career adaptability among college students: a chain intermediary model*,” deeply analyzed career adaptability among college students in China. Data from 1,182 college students were gathered using the Meaning in Life Questionnaire, the Simplified Coping Style Questionnaire, the Adult General Hope Scale, and the Career Adapt-Abilities Scale. The results showed a significant positive correlation among wellbeing (meaning in life), positive coping styles, hope, and career adaptability. Positive coping styles and hope play a separate mediating role and a chain mediating role. Furthermore, positive coping styles and increased levels of hope contribute to the development of career adaptability among college students.

Some studies specifically analyzed the role of planfulness and flexibility on wellbeing. Planfulness refers to an individual's tendency to be future-oriented, mentally flexible, and cognitively strategic when engaging with goals and has been shown to predict goal completion. Psychological flexibility can be conceptualized as “a generalized or higher-order capacity to adapt appropriately to situational demands while pursuing longer-term goals, allowing for the choosing of coping mechanisms that are appropriate for a specific circumstance” (Daşcı et al. p. 4).

Ameden et al., in the article, “*The role of planfulness for well-being, stress and, goal disruption, during COVID-19*”, investigated the relationships among planfulness, goal disruption, stress, and psychological wellbeing during the COVID-19 pandemic, which served as a unique setback context. They measured these constructs using the planfulness scale, an *ad-hoc* survey item probing goal disruption in the pandemic, the perceived stress scale, and the Warwick–Edinburgh Mental Wellbeing Scale, respectively. Participants were university students in this study as well (*N* = 174; mean age 23.03, SD: 4.37; 77% women). Higher planfulness predicted lower goal disruption, lower stress, and higher wellbeing during the pandemic, extending its benefits beyond the goal domain. High levels of planfulness did not protect against goal disruption among those participants in which the self-reported personal impact of the pandemic was the highest. Differences in goal disruption across levels of planfulness were constrained to lower reported pandemic impact. However, the differences in psychological wellbeing and stress by levels of planfulness were retained even when self-reported perceptions of personal pandemic impact were high. More planful students maintained lower stress and higher psychological wellbeing than their less planful peers across levels of adversity. These findings suggest that even in extremely difficult contexts in which planfulness does not protect against goal disruption, it still confers personal benefits in terms of psychological health and wellbeing.

Daşcı et al. from Turkey, in “*The relationship between tolerance for uncertainty and academic adjustment: the mediating role of students' psychological flexibility during COVID-19*,” analyzed the relationship between tolerance for uncertainty and academic adjustment defining the mediating role of students' psychological flexibility during COVID-19. Uncertainty was detected in several studies as one of the severe effects of COVID-19 (Baker et al., 2020; Zavras, 2021; Andrews et al., 2023) and here the authors deepen the role of flexibility in contrasting uncertainty issues among Turkish university students during the pandemic.

In this study, 388 university students completed five questionnaires—the Intolerance of Uncertainty Scale, the Acceptance and Action Questionnaire II, the Academic Self Efficacy Scale, the Educational Stress Scale, and the Online Self-Regulatory Learning Scale. Additionally, as indicators of students' academic adjustment, perceived academic performance (ranging from 1 to 10) and their last academic grade point average before and during the pandemic were also collected. Academic adjustment is seen as academic wellbeing and equated with a sense of achievement, freedom from stress, and self-efficacy.

The results indicated that psychological flexibility played a complete mediating role between the intolerance of uncertainty and academic adjustment. Therefore, research findings showed that psychological flexibility is a very important strength for university students to maintain their academic adjustment in stressful times.

The changes in the levels of wellbeing over the years were also addressed. Diao et al., in the article “*Emotions, COVID-19 related thoughts and satisfaction with life during the critical period from control to relaxation*,” investigated the psychological wellbeing of the general population during China's transition period from strict control measures to relaxed policies in COVID-19 prevention and control, as well as the impact of COVID-19-related thoughts on emotion and life satisfaction during the widespread infections. They conducted a cross-sectional study involving 1,578 participants who were assessed for positive and negative emotions, thoughts about COVID-19, and satisfaction with life. The findings revealed that those who had been infected with COVID-19 had lower levels of positive emotions compared to those who were uninfected. In this significant historical period, maintaining a positive perspective on COVID-19 and its management becomes paramount in enhancing, as an intermediate instance, the emotional wellbeing, life satisfaction, and overall wellbeing of individuals.

The preliminary aim of us, as guest editors, had been to explore the effects of COVID-19 and the perception of climate change on emotional life and overall wellbeing together with the perception of the war in Ukraine, along with other armed conflicts in other parts of the world, on people's emotions and experiences, and their ideas about future world prospects. These goals were, on the contrary, hard to explore. Röhrle (2024)'s article, recently published, remains the most significant contribution to the topic. It is an extensive systematic review through a community psychology lens, introducing psychological research from an ecological and multidisciplinary perspective. The author highlights how critical community psychology, unlike critical psychology, is based on evidence-based literature; within an ecological model, it interacts with broader social sciences, earth sciences, and medicine. It is therefore a critical perspective through which community psychology addresses the new “unsolvable” (wicked) problems of the world, seeking to propose intervention strategies that go beyond the acquirement of individual wellbeing (Dohrenwend, 1978; Arcidiacono, 2024).

Fedi (2023) also considers how climate and environmental traumas affect wars, migration, displacement, birthrates, and relationships and thus underlie many of the vicissitudes of the planet Earth. In fact, these are events that affect individual and collective wellbeing, which psychology cannot fail to consider when dealing with individual and collective histories.

In this Research Topic, however, contributions are at the intersection of individual responses and social interventions: two Chinese studies indeed focus on planfulness, tolerance, and hope. The attention they gave to some specific individual variables in contrasting social plagues, such as planfulness and susceptibility, is very innovative. Psychological flexibility and the role of hope are at stake in light of the relevant research and recommendations for further research and implications are provided.

Regarding further indications at the multidimensional social level, results are, however, not very encouraging. A comprehensive social perspective to identify problems and design future interventions and organizational issues to overcome them was only partially addressed in our Research Topic. Indeed, an Italian study “*COVID-19, people with disabilities, and the italian government recovery: investigating the impact and promoting psychological resources to prevent future emergencies*” investigated how to promote psychological resources to prevent future emergencies (Camussi et al.), but the study is very exploratory. The COVID-19 health emergency aggravated and amplified problems, and this research investigated the incidence of psychological distress and the role of psychological resources for people with disabilities in the aftermath of the pandemic, showing a strong relationship between the levels of psychological resources and life satisfaction during the COVID-19 pandemic. The research highlights the extension of this period's impacts on this population's psychological wellbeing and amplifies the urgent call for action and further research to preserve and enhance people's wellbeing.

Cross-cultural research about these crises remains to be conducted; probably in the coming years, we will learn more from systematic research addressing the COVID-19 recovery worldwide.

There is, however, strong evidence regarding the role of uncertainty in individual and social wellbeing. At the same time, the role of hope is assuming a significant role. Both constructs are only, in a large sense, part of psychological research (Scioli and Biller, 2009) and have only recently been introduced in a community psychology approach (Di Napoli et al., 2019; Arcidiacono et al., 2022). They need to receive more attention as Research Topics and in preventative interventions aimed at the co-construction of wellbeing and its empowerment.
